# Phase Equilibria in Systems Involving the Rare Earth Oxides. Part III. The Eu_2_O_3_−In_2_O_3_ System

**DOI:** 10.6028/jres.065A.044

**Published:** 1961-10-01

**Authors:** S. J. Schneider

## Abstract

The equilibrium phase diagram was determined for the Eu_2_O_3_−In_2_O_3_ system. An induction furnace, having an iridium crucible as the heating element (susceptor), was used to establish the solidus and liquidus curves. The 1:1 composition melts congruently at 1745 ± 10 °C. Melting point relations suggest that the 1:1 composition is a compound with solid solution extending both to 31 mole percent In_2_O_3_ and 71 mole percent In_2_O_3_. The compound is pseudohexagonal with *a*_H_ = 3.69 A and *c*_H_ = 12.38 A. Isostructural phases also occur in the 1:1 mixtures of both Gd_2_O_3_ and Dy_2_O_3_ with In_2_O_3_. The melting points of Eu_2_O_3_ and In_2_O_3_ were determined to be 2,240 ± 10 °C and 1910 ± 10 °C respectively. A eutectic occurs in the Eu_2_O_3_−In_2_O_3_ system at 1,730 °C and about 73 mole percent In_2_O_3_. The indicated uncertainties in the melting points are conservative estimates of the overall inaccuracies of temperature measurement.

## 1. Introduction

This paper is the third in a series concerning phase equilibria in systems involving the trivalent rare earth oxides. The first paper [1]^1^ described the polymorphic forms of these oxides. The second [2] dealt with solid state reactions between these materials. This investigation reports results obtained in a complete study of one specific system, Eu_2_O_3_−In_2_O_3_.

Two polymorphic forms of Eu_2_O_3_ have been reported [1]. The stable modification is monoclinic having a B-type^2^ rare earth oxide structure. The other form is metastable having the cubic C-type (Tl_2_O_3_–type) rare earth oxide structure. The transformation from the C- to the B-type occurs above about 1,075 °C and is apparently irreversible. The melting point of Eu_2_O_3_ has been reported as 2,050 °C [3].

Although In_2_O_3_ is not a member of the lanthanide series of rare earth oxides, it does have the cubic C-type structure. Indium sesquioxide becomes somewhat volatile at elevated temperatures. It has been reported to volatilize predominantly by decomposition to the gaseous elements at temperatures of 677 °C [4] and 850 °C [5]. However, as shown by the present investigation, volatilization of In_2_O_3_ does not become significant until temperatures in excess of 1,350 °C are reached and maintained for several hours. Goldschmidt et al., [6] reported the melting point of In_2_O_3_ to be over 2,000 °C.

Because of the relatively high melting points encountered in this system, induction heating was employed exclusively for the determination of the solidus and liquidus curves.

## 2. Materials

The starting materials used in this study were found by general qualitative spectrochemical analysis to have the following impurities:

Eu_2_O_3_—Ba, Ca, Er, and Si; each present in amounts less than about one hundredth percent. Cr, Cu, Fe, Mg, and Ni; each present in amounts less than about one thousandth percent.
  As and Yb; perhaps present.
In_2_O_3_—Al, Ca, Cu, Fe, Mg, Ni, Pb, and Si; each present in amounts less than about one hundredth percent.
  Er; present in amounts less than about one thousandth percent.
  Ag and Mn; each present in amounts less than about one ten thousandth percent.
  Tl; perhaps present.

## 3. Experimental Procedure

Specimens were prepared from 1 g batches of various combinations of Eu_2_O_3_ and In_2_O_3_. Calculated amounts of each oxide, corrected for ignition loss at 800 °C, were weighed to the nearest milligram. Each batch was mechanically mixed, pressed at 10,000 lb/in._2_ into a ⅜ in. diam pellet and fired on platinum foil in a muffle furnace at 800 °C for about 20 hr. The specimens were then ground in an agate mortar, remixed, again pressed into pellets, and then fired at 1,350 °C for 6 hr.

Following the preliminary heat treatments, the specimens were ground, sealed in Pt tubes and fired at various temperatures for different periods of time in a platinum alloy quench furnace. The Pt tubes containing the specimens were quenched into ice water and then examined for evidence of leaks. If leakage occurred, as indicated by small pin holes in the tube or by the presence of water in the specimen, the results were discarded and the experiment repeated.

Temperatures in the quench furnace, controlled to ±5 °C, were measured with a platinum-platinum-10-percent-rhodium thermocouple. Inasmuch as Pt thermocouples deteriorate rapidly above 1,400 °C, the temperatures recorded for all quenching experiments are only accurate to about ±10 °C.

All quenched specimens were examined by X-ray diffraction techniques at room temperature using a high-angle recording Geiger-counter diffractometer and Ni-filtered Cu radiation. Equilibrium was assumed to have been attained when the X-ray pattern showed no change after successive heat treatments of a specimen or when the data were consistent with the results from a previous set of experiments.

It was necessary, in mixtures containing greater than 50 mole percent In_2_O_3_, to heat treat the specimen for a relatively long time before equilibrium was even approached. The Pt tubes invariably failed due to the high internal pressure. Therefore the equilibrium phases had to be extrapolated from the results obtained from a series of short-time heat treatments.

An induction furnace, having as the heating element (susceptor) a small iridium crucible, was used exclusively to determine all solidus and liquidus temperatures. Temperatures in excess of 2,300 °C were easily attained with this furnace. The crucible with cover was formed by powder metallurgical techniques and had the following overall nominal dimensions; height, ¾ in.; outer diameter, ½ in.; and wall thickness, 1/16 in. A small fragment of a 1,350 °C calcined specimen was placed on a small iridium button which in turn was set inside the crucible. Temperatures were controlled manually and were measured with an optical pyrometer sighted through a calibrated 45° glass prism and a 116 in. diam hole in the crucible cover.

Nearly perfect blackbody conditions were obtained with this arrangement. The specimen generally could not be seen. All heatings were performed in a normal atmosphere. Vaporization of IrO_2_ and/or Ir apparently did not affect the specimen in any way. Solid state reaction between the specimen and these materials was never indicated by the X-ray patterns of the various specimens.

The crucible was heated very quickly at first and then more slowly as the desired temperature was approached. When the appropriate temperature was reached and maintained for about 15 sec, the furnace was turned off. The temperature of the crucible dropped below red heat in less than 1 min. The entire heating and cooling cycle consumed about 10 min. or less. The cooled specimen was examined visually with the binocular microscope for evidence of melting. The solidus temperature was indicated by slight rounding of the corners of the specimen. The liquidus temperature, usually more difficult to determine, was indicated by the formation of an essentially flat button with no sharp or irregular corners.

The apparent temperatures indicated by the pyrometer were dependent upon the physical setup of the equipment and geometry of the crucible. The temperature measuring system was calibrated against Au, Pt, and Rh (set on alumina) and as expected, each metal appeared to melt at a temperature lower than its reported value. The deviations between apparent and actual melting points were accounted for in part by errors caused by the pyrometer and prism. After correcting for the pyrometer and prism errors, the deviations amounted to 3 °C at 1,063 °C (Au), 11 °C at 1,769 °C (Pt), and 13 °C at 1,960 °C (Rh). A plot of temperature deviation against apparent temperature results in a linear curve which was used throughout this investigation to correct all observed temperature to a precision of at least ±5 °C. The average emissivity for the three calibration points was 0.953 with a range of ±0.012, which is smaller than the inaccuracy of the pyrometer. For these reasons the calibration curve was extended above the last calibration point (1,960 °C) with reasonable expectancy that the emissivity will not change.

The reasons for the small deviation from blackbody conditions are unknown at this time. At first it was thought possible that part of the deviation was due to IrO_2_ and/or Ir vapor. This assumption was disproved, however, by an experiment in which the filament of a standard pyrometer lamp showed no temperature deviation when sighted on through vapor of IrO_2_ and/or Ir. The experiment consisted of sighting through a hollow iridium cylinder on the lamp filament which was maintained at a constant temperature. The temperature of the filament was determined with an optical pyrometer both before and during the heating of the cylinder. The cylinder was maintained at various temperatures, ranging between about 1,000 °C and the melting point of iridium. Within the limits of the precision of the pyrometer, any absorption of radiation by the vapor of IrO_2_ and/or Ir produced by the heated cylinder would have been easily detected. An imperfect crucible (variation in density) is the most probable explanation for the deviation from blackbody conditions. These imperfections could cause nonuniform heating and thus result in deviation from blackbody conditions. However, nonuniform heating was not detected by the optical pyrometer when sighted on various parts of the crucible.

Because of the tendency for In_2_O_3_ to volatilize, it is probable that the measured melting point might actually represent the melting point of some mixture lower in In_2_O_3_ content than the starting composition. This error was greatly minimized by the rapid heating technique employed. Extensive volatilization of In_2_O_3_ was easily detected by a scoriaceous appearance of the specimen. It should be emphasized that the problem of volatilization of In_2_O_3_ was not as serious as had been anticipated. Weight loss data on In_2_O_3_ after heat treatment at 1,350 °C for 6 hr in air showed that a maximum compositional error of only about 0.25 mole percent would result for the 25 mole percent Eu_2_O_3_:75 mole percent In_2_O_3_ mixture. The error for the 50:50 composition would be even less. Furthermore, weight loss data on In_2_O_3_ heated to high temperatures in a manner similar to the melting point experiments indicated that additional compositional errors would not be excessive. For specimens heated to 1,730 °C and 1,900 °C the compositional error (calculated) for the 75:25 mixture would be about 0.34 mole percent and 1.02 mole percent respectively. For this system these possible errors were probably reduced even further by solid state reaction which would tend to inhibit the loss of In_2_O_3_. Nevertheless, considering the various errors, it is estimated that the solidus and liquidus temperatures up to about 2,000 °C, as recorded, are accurate to ±10 °C and ±20 °C, respectively. Exact estimates of the accuracy for temperatures between 2,000 and 2,300 °C are not available because of lack of calibration data. However, it is believed that the accuracy of temperatures in this range is no worse than the values previously stated for lower temperatures provided that the calibration curve can be extended above 2,000 °C. The reproducibility of results was found to be better than ±5 °C.

## 4. Results and Discussion

**Figure 1:**
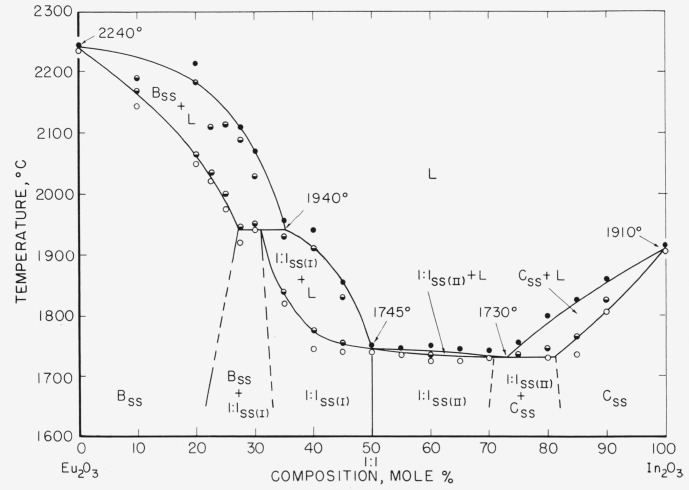
*Phase equilibrium diagram for the system* Eu_2_O_3_−In_2_O_3_. B—Eu_2_O_3_, having the B-type rare earth oxide structure. C—In_2_O_3_ having the C-typo rare earth oxide structure. 1:1—Eu_2_O_3_, · In_3_O_3_ compound. L—liquid ss—solid solution. ○—no melting. ◒—partial melting. ●—completely melted.

The equilibrium phase diagram for the Eu_2_O_3_−In_2_O_3_ system is given in figure 1. It was constructed from the data listed in tables 1 and 2. Only the more significant data are shown on the diagram. The difficulty in obtaining equilibrium in many mixtures as well as the inability to quench solid solutions without causing exsolution made it necessary to rely on the shape of the solidus and liquidus curves to determine the different subsolidus boundary limits.

The melting point of Eu_2_O_3_ was found to be 2,240 ± 10 °C. The X-ray pattern of melted Eu_2_O_3_ was very diffuse and therefore difficult to interpret. It showed essentially only those diffraction peaks attributable to the B-type structure. Wisnyi and Pijamowski [3], using a tungsten strip furnace in a hydrogen atmosphere, determined the melting point of Eu_2_O_3_ as 2,050 ± 30 °C. One possible explanation for the large difference in the two melting points is that Eu_2_O_3_ probably partially reduces to EuO in hydrogen.

Solid solution of In_2_O_3_ in Eu_2_O_3_ (B_ss_) occurs from 0 to about 27 mole percent In_2_O_3_ at the solidus. X-ray patterns of various quenched specimens in this region contained two diffraction peaks *(d*=2.9079 A and 2.7684 A) which normally are not associated with those of the B-type structure. The occurrence of the two extraneous peaks was not a consistent phenomena, thus suggesting that the peaks represent a metastable second phase. The X-ray patterns of heat treated rare earth oxides commonly show diffraction peaks attributable to rare earth oxide structures other than those of the stable phase [1]. The two extra peaks occur at *d*-spacings that correspond closely to the spacings of the two strongest reflections, 002 and 101, predicted for Eu_2_O_3_ having the hexagonal A-type (La_2_O_3_-type) structure. If this is the correct interpretation Eu_2_O_3_ apparently has a metastable A-type polymorph, not previously reported.

The phase diagram (fig. 1) was drawn to indicate a congruently melting (1,745 ± 10 °C) 1:1 compound with solid solution extending on both sides of the equimolar composition. This interpretation was necessitated by the melting point data which indicated that a sharp inflection occurred in the liquidus curve at about the 1:1 composition. An alternate interpretation might be a continuous region of solid solution with a minimum close to the 65:35 composition.

The subject of a compound occurring with solid solution on both sides is amply discussed by Zernike [8]. An example of this type of phenomenon was reported by Bowen et al., [9] in the system, Fe_2_-SiO_4_−Ca_2_SiO_4_. It should be noted that if a compound occurs in the middle of a solid solution region, a plot of some intensive property versus composition should show a sharp change in slope at the composition of the compound. Several experiments were performed in an attempt to find other changes in intensive properties besides the melting points. Specimen ranging in composition from 54Eu_2_O_3_: 46In_2_O_3_ to 46Eu_2_O_3_:54In_2_O_3_ (in one mole percent increments) were prepared and quenched from 1,650 °C. X-ray analysis showed that specimens containing greater than 50 mole percent Eu_2_O_3_ were composed of a mixture of phases, 1:1 and B_ss_. Those containing 50 mole percent or less of Eu_2_O_3_ were single phase 1:1_ss_. The inability to quench in the solid solution on the high Eu_2_O_3_ portion makes it impossible to observe a sharp change in intensive properties. It is interesting to observe that it is only the melting point data which indicates solid solution of the 1:1 compound at compositions containing greater than 50 mole percent Eu_2_O_3_. For compositions containing less than 50 mole percent Eu_2_O_3_, it is the solid state data which gives this indication. This observation, in a sense, would suggest a sharp change in intensive properties. The solid solution areas on either side of Eu_2_O_3_·In_2_O_3_ in figure 1 are labeled as 1:1_ss(i)_ and 1:1_ss(II)_ in order to delineate the expected change in intensive properties.

The single phase 1:1 solid solution areas extend from about 31 to 50 mole percent In_2_O_3_ and from 50 to about 71 mole percent In_2_O_3_ at the solidus. Apparently exsolution was extensive. Solid solution of Eu_2_O_3_ in the 1:1 compound amounts to less than one mole percent at room temperature.

The X-ray pattern of Eu_2_O_3_·In_2_O_3_ is given in table 3 along with the patterns of the 1:1 mixtures of Gd_2_O_3_−In_2_O_3_ and Dy_2_O_3_−In_2_O_3_^2^. All three of these phases appear to be isostructural. The structure-type has not been previously reported. The latter two phases were prepared from equimolar mixtures which had been heat treated at 1,600 °C in sealed Pt tubes.

After further heat treatment, at 1,650 °C, only the 1:1 Dy_2_O_3_−In_2_O_3_ specimen decomposed to a mixture of B-type and C-type solid solutions. With the exception of one diffraction peak (*d*=1.9317 A), the pattern of Eu_2_O_3_·In_2_O_3_ was indexed on a hexagonal basis with *a_H_* and *c_H_* equal to 3.69 A and 12.38 A, respectively. The other two listed patterns could not be similarly indexed with the same degree of agreement between observed and calculated 1/*d*^2^ values. The marked difference between the patterns of 1:1 Gd_2_O_3_−In_2_O_3_ and 1:1 Dy_2_O_3_−In_2_O_3_, and that of Eu_2_O_3_·In_2_O_3_ is that the corresponding 101 and 202 reflections of the former are doublets. However, the unindexed peak (*d*=1.9317 A) of the Eu_2_O_3_·In_2_O_3_ pattern is present in the other two patterns. Apparently all three phases possess a lower symmetry than hexagonal. The listed parameters of Eu_2_O_3_·In_2_O_3_ are therefore based on a pseudocell.

The occurrence of a perovskite-type compound (orthorhombic) was prevalent in mixtures that contained greater than 50 mole percent In_2_O_3_. Extended heat treatment of the 55 and 60 mole percent In_2_O_3_ specimens caused the complete disappearance of the perovskite-type compound, thus suggesting that it was metastable having no equilibrium position on the phase diagram. However, for specimens higher in In_2_O_3_ content than 60 mole percent, the perovskite-type phase was never completely eliminated, only reduced in amount. Failure to eliminate the perovskite phase in these specimens arose from experimental difficulties. A phase transformation of Eu_2_O_3_·In_2_O_3_ to the perovskite-type structure is not considered probable, although this must be considered as a possibility. Throughout the entire temperature range studied for the 1:1 mixture, this perovskite structure was never identified in the X-ray patterns of the various specimens.

The formation of the perovskite-type structure was not unexpected. This structure occurs in the Sm_2_O_3_−In_2_O_3_ system [10] as a stable 1:1 phase. Samarium sesquioxide (Sm_2_O_3_) has just slightly larger unit cell dimensions than Eu_2_O_3_. Evidently Sm^+3^ is the smallest rare earth ion that will form a stable perovskite-type structure with In^+3^.

Because of experimental difficulties, all attempts to locate the exact limits of the two phase area 1:1_ss(II)_ + C_ss_ (In_2_O_3ss_) by solid state reaction were unsuccessful. It is estimated from melting point data to extend from about 71 to 81 mole percent In_2_O_3_ at the solidus. The eutectic occurs at 1,730 °C and about 73 mole percent In_2_O_3_.

The melting point of In_2_O_3_ was determined to be 1,910 ± 10 °C. X-ray patterns of In_2_O_3_ heated to 1,905 °C and 1,915 °C showed only the diffraction peaks identifiable with cubic In_2_O_3_. Specimens heated to 1,985 °C and 2,000 °C completely vaporized.

## 5. Summary

The equilibrium phase diagram for the Eu_2_O_3_−In_2_O_3_ system was determined from a study of solid state reactions and melting point relations. The quenching technique was employed for subsolidus experiments. An inductively heated iridium crucible was used exclusively for the determination of solidus and liquidus temperatures. Phases were identified by examination of X-ray diffraction patterns.

Eu_2_O_3_ and In_2_O_3_ were found to melt at 2,240 ± 10 °C and 1,910 ± 10 °C respectively. The presence of extraneous diffraction peaks in X-ray patterns of several specimens containing Eu_2_O_3_ solid solutions indicated that Eu_2_O_3_ may have a metastable polymorph of the A-type rare earth oxide structure.

A sharp inflection in the liquidus curve suggested that a Eu_2_O_3_·In_2_O3 compound exists in the system with solid solution extending on both sides of the equimolar composition. The compound melts congruently at 1,745 ± 10 °C. Its X-ray pattern was indexed on the basis of a pseudo-hexagonal cell with *a_H_*=3.69 A and *c_H_*=12.38 A. Isostructural phases have been found at the equimolar mixtures in the Gd_2_O_3_−In_2_O_3_ and Dy_2_O_3_−In_2_O_3_ systems. A eutectic occurs in the Eu_2_O_3_−In_2_O_3_ system at 1,730 °C and about 73 mole percent In_2_O_3_.

## Figures and Tables

**Table 1:** Experimental data for compositions in the Eu_2_O_3_−In_2_O_3_ system

Composition		Heat treatment^2^		X-ray diffraction analyses^3^	Remarks
Eu_2_O_3_	In_2_O_3_^1^	Temp.	Time		
					
*Mole %*	*Mole %*	°C	*hr*		
100	0	1,500	1.5	B^5^	
		2,245	−(4)	B	Specimen melted.
90	10	1,600	6	B_ss_5	
		1,650	1.5	B_ss_	
85	15	1,650	1.5	B_ss_	
80	20	1,600	1	B_ss_	
		1,600	6	B_ss_^5^	
		1,650	1.5	B_ss_	Extraneous X-ray peak at *d*=2.9079 A.
77.5	22.5	1,650	1.5	B_ss_	Extraneous X-ray peaks at *d*=2.9079 A and 2.7684 A.
75	25	1,600	6	B_ss_+1:1^5^	
		1,600	1.5	B_ss_+1:1	Extraneous X-ray peak at *d*=2.9079 A.
70	30	1,600	6	B_ss_+1:1^5^	
		1,600	1.5	B_ss_+1:1	
65	35	1,600	2	1:1+B_ss_	
60	40	1,600	6	1:1+B_ss_^5^	
55	45	1,600	2	1:1+B_ss_	
54	46	1,650	2	1:1+B_ss_	
53	47	1,650	2	1:1+B_ss_	
52	48	1,650	2	1:1+B_ss_	
51	49	1,650	2	1:1+B_ss_	
50	50	1,350	6	1:1^5^	
		1,600	6	1:1^5^	
		1,650	6	1:1^5^	
		1,650	2	1:1	
		1,730	.5	1:1+B_ss_	Specimen lost In_2_O_3_. Heated in Ir crucible, non-equilibrium for equimolar mixture.
49	51	1,650	2	1:1_ss_	
48	52	1,650	2	1:1_ss_	
47	53	1,650	2	1:1_ss_	
46	54	1,650	2	1:1_ss_	
45	55	1,650	1	1:1_ss_+P	Nonequilibrium.
			2^6^	1:1_ss_	
40	60	1,600	1	P+1:1_ss_+C_ss_	Nonequilibrium.
		1,650	3^6^	1:1_ss_+P	Nonequilibrium.
		1,650	2	1:1_ss_	
35	65	1,650	3	P+1:1_ss_+C_ss_	Nonequilibrium.
		1,650	2^6^	1:1_ss_+P	Nonequilibrium.
30	70	1,650	2	P+C_ss_+1:1_ss_	Nonequilibrium.
		1,650	3^6^	1:1_ss_+P	Nonequilibrium.
	100	1,650	6	C	
		1,905	−(4)	C	
		1,915	−(4)	C	Specimen melted.

1 Results of experiments on compositions intermediate between 70 and 100 mole percent In_2_O_3_ are not included. Pt tube always failed during beat treatments, giving erroneous results.

2 All specimens calcined at 800 °C for 20 hr and 1,350 °C for 6 hr prior to listed heat treatment. Unless otherwise noted, all specimens were quenched from indicated temperature.

3 The phases identified are given in order of relative amount present at room temperature.

B—Eu_2_O_3_ having B-type rare earth oxide structure.

1:1—Eu_2_O_3_·In_2_O_3_ compound.

C—In_2_O_3_ having C-type rare earth oxide structure.

P—1:1 compound having perovskite-type structure.

ss—solid solution.

4 Melting point experiment; specimen held at temperature for 15 sec; specimen not quenched.

5 Specimen not quenched.

6 Additional heat treatment of previous specimen.

**Table 2:** Melting characteristics of the *Eu_2_O_3_−In_2_O_3_* system^1^

Composition			Results			Composition			Results		
Eu_2_O_3_	In_2_O_3_	Temp.	No melting	Partial melting	Complete melting	Eu_2_O_3_	In_2_O_3_	Temp.	No melting	Partial melting	Complete melting
											
*Mole %*	*Mole %*	*°C*				*Mole %*	*Mole %*	*°C*			
100	0	2,110	X	……….	……….	60	40	1,745	X	……….	……….
		2,175	X	……….	……….	……….	……….	1,775	……….	X	……….
		2,235	X	……….	……….	……….	……….	1,800	……….	X	……….
		2, 245	……….	……….	X	……….	……….	1,805	……….	X	……….
		2, 280	……….	……….	X	……….	……….	1,825	……….	X	……….
		2,330	……….	……….	X	……….	……….	1,840	……….	X	……….
90	10	2,105	X	……….	……….	……….	……….	1,860	……….	X	……….
		2,145	X	……….	……….	……….	……….	1,880	……….	X	……….
		2,170	……….	X	……….	……….	……….	1,910	……….	X	……….
		2,190	……….	X	……….	……….	……….	1,940	……….	……….	X
80	20	1,920	X	……….	……….	55	45	1,740	X	……….	……….
		1,960	X	……….	……….	……….	……….	1,755	……….	X	……….
		1,970	X	……….	……….	……….	……….	1,780	……….	X	……….
		1,990	X	……….	……….	……….	……….	1,810	……….	X	……….
		2,010	X	……….	……….	……….	……….	1,830	……….	X	……….
		2,050	X	……….	……….	……….	……….	1,855	……….	……….	X
		2,065	……….	X	……….	……….	……….	1,880	……….	……….	X
		2,095	……….	X	……….	……….	……….	1,915	……….	……….	X
		2,130	……….	X	……….	50	50	1,730	X	……….	……….
		2,170	……….	X	……….	……….	……….	1,740	X	……….	……….
		2,185	……….	X	……….	……….	……….	1,750	……….	……….	X
		2,215	……….	……….	X	……….	……….	1,760	……….	……….	X
		2,230	……….	……….	X	……….	……….	1,705	……….	……….	X
77.5	22.5	1,940	X	……….	……….	……….	……….	1,780	……….	……….	X
		2,020	X	……….	……….	45	55	1,735	X	……….	……….
		2,035	……….	X	……….	……….	……….	1,745	……….	……….	X
		2,110	……….	X	……….	40	60	1,715	X	……….	……….
75	25	1,905	X	……….	……….	……….	……….	1,725	X	……….	……….
		1,965	X	……….	……….	……….	……….	1,735	……….	X	……….
		1,975	X	……….	……….	……….	……….	1,750	……….	……….	X
		2,000	……….	X	……….	……….	……….	1,795	……….	……….	X
		2,030	……….	X	……….	35	65	1,725	X	……….	……….
		2,040	……….	X	……….	……….	……….	1,745	……….	……….	X
		2,045	……….	X	……….	30	70	1,680	X	……….	……….
		2,055	……….	X	……….	……….	……….	1,695	X	……….	……….
		2,060	……….	X	……….	……….	……….	1,705	X	……….	……….
		2,085	……….	X	……….	……….	……….	1,720	X	……….	……….
		2,115	……….	X	……….	……….	……….	1,725	X	……….	……….
72.5	27.5	1,860	X	……….	……….	……….	……….	1,730	X	……….	……….
		1,920	X	……….	……….	……….	……….	1,735	……….	X	……….
		1,945	……….	X	……….	……….	……….	1,740	……….	……….	X
		1,955	……….	X	……….	……….	……….	1,745	……….	……….	X
		1,970	……….	X	……….	……….	……….	1,755	……….	……….	X
		1,985	……….	X	……….	……….	……….	1,780	……….	……….	X
		2,010	……….	X	……….	……….	……….	1,810	……….	……….	X
		2,025	……….	X	……….	……….	……….	1,840	……….	……….	X
		2,040	……….	X	……….	25	75	1,675	X	……….	……….
		2,070	……….	X	……….	……….	……….	1,735	……….	X	……….
		2,090	……….	X	……….	……….	……….	1,755	……….	……….	X
		2,110	……….	……….	X	……….	……….	1,770	……….	……….	X
70	30	1,810	X	……….	……….	……….	……….	1,780	……….	……….	X
		1,825	X	……….	……….	……….	……….	1,795	……….	……….	X
		1,840	X	……….	……….	……….	……….	1,805	……….	……….	X
		1,895	X	……….	……….	……….	……….	1,845	……….	……….	X
		1,900	X	……….	……….	……….	……….	1,855	……….	……….	X
		1,915	X	……….	……….	20	80	1,730	X	……….	……….
		1,940	X	……….	……….	……….	……….	1,745	……….	X	……….
		1,950	……….	X	……….	……….	……….	1,800	……….	……….	X
		1,965	……….	X	……….	15	85	1,735	X	……….	……….
		1,970	……….	X	……….	……….	……….	1,765	……….	X	……….
		1,975	……….	X	……….	……….	……….	1,800	……….	X	……….
		2,000	……….	X	……….	……….	……….	1,825	……….	……….	X
		2,030	……….	X	……….	10	90	1,805	X	……….	……….
		2,070	……….	……….	X	……….	……….	1,825	……….	X	……….
65	35	1,805	X	……….	……….	……….	……….	1,860	……….	……….	X
		1,820	X	……….	……….	0	100	1,820	X	……….	……….
		1,840	……….	X	……….	……….	……….	1,905	X	……….	……….
		1,850	……….	X	……….	……….	……….	1,915	……….	……….	X
		1,855	……….	X	……….	……….	……….	1,985	……….	……….	(2)
		1,870	……….	X	……….	……….	……….	2,000	……….	……….	(2)
		1,880	……….	X	……….						
		1,885	……….	X	……….						
		1,930	……….	X	……….						
		1,955	……….	……….	X						

1 Specimens calcined at 800 °C for 20 hr and 1350 °C for 6 hr prior to melting point determination.

2 Specimens completely vaporized.

**Table 3:** X-ray diffraction powder data for Eu_2_O_3_·In_2_O_3_ and equimolar mixtures of Gd_2_O_3_−In_2_O_3_ and Dy_2_O_3_−In_2_O_3_ (CuK_a_ radiation)

Eu_2_O_3_*·*In_2_O_3_					1:1 Gd_2_O_3_−In_2_O_3_		1:1 Dy_2_O_3_−In_2_O_3_	
*hkl*^a^	*d*^b^	1/*d*^2^		*I/I*_0_^d^	*d*^b^	*I/I*_0_^d^	*d*^b^	*I/I*_0_^d^
		*obs*	*cal*^c^					
								
	*A*				*A*		*A*	
100	3.1963	0.0979	0.0979	13	3.1739	16	3.1443	12
101	3.0940	.1045	.1044	100	$\left\{ \begin{array}{l} 3.0847\\ 3.0732 \end{array}\right.$	55 60	3.0701 3.0475	30 73
102	2.8383	.1241	.1240	100	2.8219	100	2.8004	100
103	2.5258	.1567	.1566	12	2.5135	18	2.4953	13
104	2.2222	.2025	.2022	7	2.2139	15	2.1968	12
105	1.9560	.2614	.2610	8	1.9489	20	1.9371	15
(e)	1.9317	.2680	……	3	1.9270	7	1.9178	6
110	1.8461	.2934	.2937	41	1.8332	35	1.8171	30
106	1.7338	.3326	.3326	17	1.7278	20	1.7172	21
201	1.5855	.3978	.3982	24	1.5759	26	1.5642	33
202	1.5475	.4176	.4177	17	$\left\{ \begin{array}{l} 1.5424\\ 1.5384 \end{array}\right.$	10 19	1.5335 1.5018	7 12

a Pseudo hexagonal Miller indices.

b Interplanar spacing.

c Based on the unit-cell parameters, *a_H_*=3.69 A, *c_H_*=12.38 A.

d Relative intensity.

e This peak docs not fit pseudo hexagonal cell.

## Footnotes

1 Figures in brackets indicate the literature references at the end of this paper.

2 This structure type has not yet been fully described in the crystallographic literature.

2 The purity of the Gd_2_O_3_ and Dy_2_O_3_ used was greater than 99.9 percent. Complete analyses are given elsewhere [2].

## References

[1] Roth RS, Schneider SJ (1960). Pt I, J Research NBS.

[2] Schneider SJ, Roth RS (1960). Pt II, J Research NBS.

[3] Wisnvi LG, Pijanowski S (1956). US Atomic Energy Comm.

[4] Brewer L (1953). Chem Rev.

[5] (1958). Handbook of Chemistry and Physics.

[6] Goldschmidt VM, Barth T, Lunde G (1925). Skrifter Norske Videnskaps-Akad Mat Natur Kl.

[7] Cromer DT, Phys J (1957). Chem.

[8] Zernike J (1955). Chemical Phase Theory.

[9] Bowen NL, Schairer JF, Posnjak E (1933). Am J Sci, 5th Ser.

[10] Roth RS (1957). J Research NBS.

